# Regulation and Role of Adiponectin Secretion in Rat Ovarian Granulosa Cells

**DOI:** 10.3390/ijms25105155

**Published:** 2024-05-09

**Authors:** Yue Zhou, Shuhao Zhang, Yurong Jia, Xi Wang, Yuning Liu, Haolin Zhang, Zhengrong Yuan, Yingying Han, Qiang Weng

**Affiliations:** 1 College of Biological Science and Technology, Beijing Forestry University, Beijing 100083, China; animalzhouyue@bjfu.edu.cn (Y.Z.); jiayurong1022@163.com (Y.J.); wangyixin808@bjfu.edu.cn (X.W.); yuningliu9@bjfu.edu.cn (Y.L.); haolinzhang@bjfu.edu.cn (H.Z.); zryuan@bjfu.edu.cn (Z.Y.); 2 State Key Laboratory of Membrane Biology, Tsinghua-Peking Center for Life Sciences, School of Pharmaceutical Sciences, Tsinghua University, Beijing 100084, China; shuhaoszhang@foxmail.com

**Keywords:** FSH, adiponectin, AdipoR1, AdipoR2, GLUTs, ovarian granulosa cells

## Abstract

Adiponectin is an important adipokine involved in glucose and lipid metabolism, but its secretion and potential role in regulating glucose utilization during ovarian development remains unclear. This study aims to investigate the mechanism and effects of follicle-stimulating hormones (FSHs) on adiponectin secretion and its following impact on glucose transport in the granulosa cells of rat ovaries. A range of experimental techniques were utilized to test our research, including immunoblotting, immunohistochemistry, immunofluorescence, ELISA, histological staining, real-time quantitative PCR, and transcriptome analysis. The immunohistochemistry results indicated that adiponectin was primarily located in the granulosa cells of rat ovaries. In primary granulosa cells cultured in vitro, both Western blot and immunofluorescence assays demonstrated that FSH significantly induced adiponectin secretion within 2 h of incubation, primarily via the PKA signaling pathway rather than the PI3K/AKT pathway. Concurrently, the addition of the AdipoR1/AdipoR2 dual agonist AdipoRon to the culture medium significantly stimulated the protein expression of GLUT1 in rat granulosa cells, resulting in enhanced glucose absorption. Consistent with these in vitro findings, rats injected with eCG (which shares structural and functional similarities with FSH) exhibited significantly increased adiponectin levels in both the ovaries and blood. Moreover, there was a notable elevation in mRNA and protein levels of AdipoRs and GLUTs following eCG administration. Transcriptomic analysis further revealed a positive correlation between the expression of the intraovarian adiponectin system and glucose transporter. The present study represents a novel investigation, demonstrating that FSH stimulates adiponectin secretion in ovarian granulosa cells through the PKA signaling pathway. This mechanism potentially influences glucose transport (GLUT1) and utilization within the ovaries.

## 1. Introduction

Adiponectin, also known as Acrp30, GBP28, or apM1, is a fat-derived hormone that plays a pivotal role in numerous physiological functions, particularly in glycolipid metabolism and insulin sensitivity [1,2,3,4,5]. Recent studies have revealed that, beyond adipose tissues, various other tissues possess the capability to independently synthesize adiponectin [6,7,8]. This growing body of evidence highlights the potential importance of locally produced adiponectin in autocrine or paracrine actions. In mammalian ovaries, adiponectin and its receptors (AdipoR1 and AdipoR2) are found in different ovarian cell types, follicular fluid, and accessory gonadal organs across species [9,10,11,12]. However, the regulatory mechanism of local adiponectin secretion in the ovary remains unclear. Studies employing adiponectin-deficient female mice have revealed disruptions in reproductive cycles, reduced egg counts, and increased follicular atresia [13,14]. Notably, extensive research has documented the close association of adiponectin with various hormones. For instance, it influences follicular development and ovarian reserve by regulating the gene expression of amphiregulin and AMHs (anti-mullerian hormones) [15,16,17]. Adiponectin deficiency disrupts the function of the hypothalamic–pituitary axis, impacting FSH (follicle-stimulating hormone) and LH (luteinizing hormone) secretion, along with a surge in LHs [18]. These findings highlight the regulatory impact of adiponectin on follicular development, ovulation, and steroidogenesis during physiological and pathological processes in the ovaries. However, the direct experimental evidence regarding the involvement of adiponectin in the regulation of glycolipid absorption or metabolism in the ovaries is still lacking.

Glucose is of paramount importance in supporting ovarian activity as it is the primary energy source for follicular development and steroid hormone synthesis [19,20,21,22]. The transport and utilization of glucose in granulosa cells are particularly essential for oocyte maturation. Since the oocyte cannot directly utilize glucose, it depends on acetate and lactate produced from glucose metabolism in the surrounding granulosa cells [23,24]. To date, 14 distinct glucose transporters (GLUTs) have been recognized for facilitating glucose diffusion across cell membranes [25]. Among these, GLUT1, 2, 3, and 4 have been detected in the ovaries, impacting ovarian glucose homeostasis and metabolism [26]. More than that, it has been demonstrated that FSH plays an intricate role in regulating the expression of GLUTs in ovarian granulosa cells [21,27,28]. The regulation of GLUT expression by FSH has also been influenced by intraovarian factors, including estrogen, insulin-like growth factor-I (IGF-I), and interleukin-1β [22,29]. In skeletal muscle, it has been proven that adiponectin enhances glucose utilization by promoting GLUT4 translocation to the plasma membrane through the activation of AMP-activated protein kinase (AMPK) or mitogen-activated protein kinase (p38 MAPK). Additionally, ovaries treated with adiponectin in the Cynopterus sphinx exhibited enhanced progesterone synthesis through the increased glucose uptake facilitated by GLUT4 and AdipoR1 [30,31]. These findings suggest that adiponectin could regulate glucose utilization, but its direct involvement in glucose homeostasis in the ovary remains unknown.

Therefore, building on previous research findings, this study aims to elucidate the regulatory mechanism of locally secreted adiponectin within ovarian granulosa cells, highlighting its pivotal role in glucose transport and utilization. Furthermore, this study seeks to clarify the complex interplay among FSH, adiponectin, and GLUT in the rat ovary, providing a theoretical foundation for investigating the involvement of adipokines in ovarian follicle development, maturation, and energy metabolism.

## 2. Results

### 2.1. FSH Stimulated Adiponectin Secretion through PKA Signaling in Rat Ovarian Granulosa Cells

**Figure 1:**
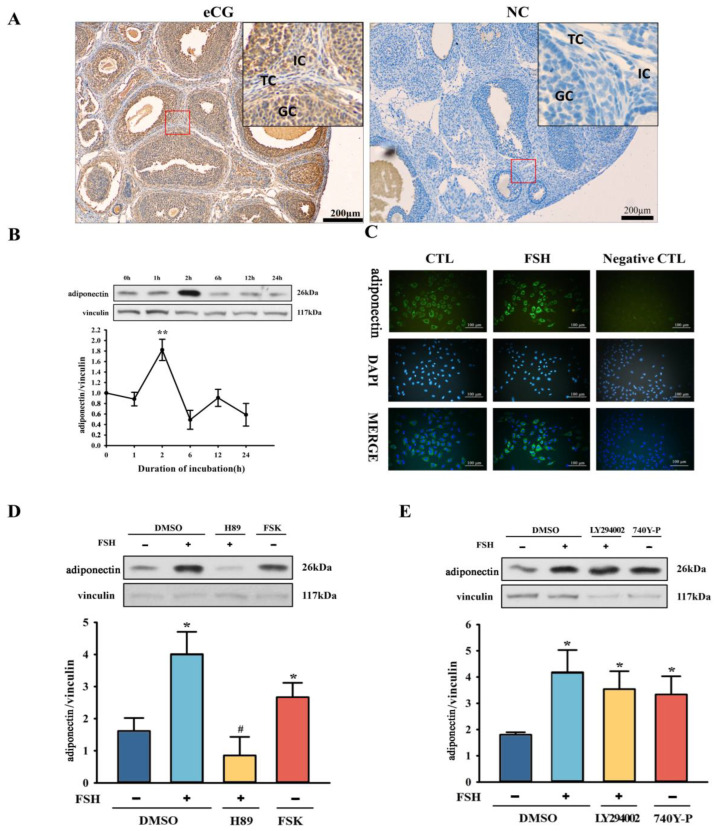
Signaling mechanisms and role of FSH on adiponectin expression in rat ovarian granulosa cells. (**A**) Protein localization of adiponectin in the ovary. (**B**,**C**) Adiponectin protein levels with the addition of FSH in vitro. Green: adiponectin; Blue: cell nucleus. (**D**,**E**) Adiponectin protein levels with the addition of the PI3K signaling pathway inhibitor and activator in vitro. +/−: represents the addition or absence of FSH. DMSO: dimethyl sulfoxide; H89: PKA inhibitor; FSK: Forskolin, PKA activator; LY294002: an inhibitor of PI3K; and 740Y-P: a potent and cell-permeable PI3K activator. NC: Negative control, the negative control group of the experiment. The error bars represent means ± SEM (*n* = 3, each group). * Statistically significant values (* *p* < 0.05; ** *p* < 0.01); # represents no significance. Scale bars: 500 μm. GC, granulosa cell; TC, theca cell; and IC, interstitial cell.

We examined the immunohistochemical localization and semi-quantitative analysis of adiponectin in rat ovaries. As shown in Figure 1A and Table 1, the immunoreactivity of adiponectin was detected in the granulosa cells, theca cells, and interstitial cells, with the highest expression observed in granulosa cells. To understand the possible role of FSH in the secretion of adiponectin in rat ovarian granulosa cells, 50 ng/mL of FSH was added into the in vitro cultured primary granulosa cells, and the protein expression of adiponectin in the cultured granulosa cells was tested at different time points (1 h, 2 h, 6 h, 12 h, and 24 h). As shown in Figure 1B, the protein expression of adiponectin in rat primary granulosa cells was significantly elevated after 2 h of FSH treatment (*p* < 0.01). Consistent with these Western blot data, the immunofluorescence study showed enhanced cytoplasmic adiponectin-positive staining in rat ovarian granulosa cells in the presence of 50 ng/mL FSH in vitro (Figure 1C).

To determine the possible signaling mechanisms involved in the FSH-regulated secretion of adiponectin in rat granulosa cells, chemical agonists and antagonists of the PI3K and PKA signaling pathways were selectively added into the in vitro cultured rat ovarian granulosa cells. As shown in Figure 1D, 10 µM of Forskolin, a PKA agonist, markedly increased adiponectin protein expression in rat granulosa cells, mimicking the effects of FSH. Conversely, the PKA signaling pathway inhibitor H89 at 10 µM significantly suppressed FSH-induced adiponectin protein expression in rat ovarian granulosa cells after 2 h of in vitro culture (Figure 1D). Unlike PKA signaling effects, the addition of 10 µM LY294002 (PI3K inhibitor) in vitro did not significantly affect adiponectin protein expression in cultured rat granulosa cells, regardless of the presence of FSH after 2 h of culture. However, after the 740Y-P (PI3K activator) treatment, the protein level of adiponectin was significantly increased (Figure 1E).

### 2.2. AdipoRon Stimulated the Protein Expression of GLUTs and Glucose Absorption in Rat Ovarian Granulosa Cells 

**Figure 2:**
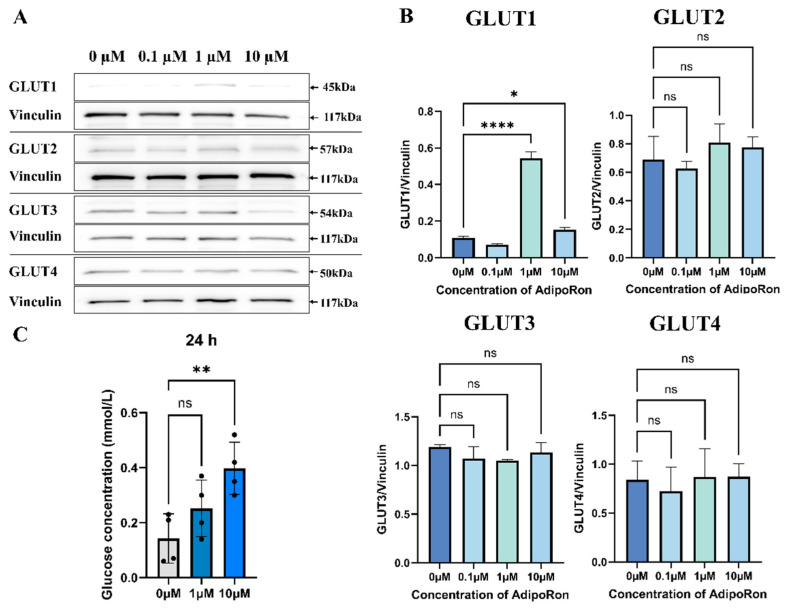
Effects of AdipoRon on the transport of glucose in rat ovarian granulosa cells. (**A**) GLUT1, GLUT2, GLUT3, and GLUT4 protein levels after 24 h of the in vitro addition of different concentrations of the adiponectin receptor agonist adipoRon treatment (0 µM, 0.1 µM, 1 µM, and 10 µM). (**B**) Histogram analysis of Western Blot gray values corresponding to changes in GLUT1, GLUT2, GLUT3, and GLUT4 protein levels after in vitro treatment with different concentrations of the adiponectin receptor agonist adipoRon (0 µM, 0.1 µM, 1 µM, and 10 µM). (**C**) Levels of glucose content in granulosa cells after 24 h of treatment with different concentrations of the adiponectin receptor agonist AdipoRon in vitro (0 µM, 1 µM, and 10 µM). The error bars represent means ± SEM (*n* = 3, each group). ns represents no significance. * Statistically significant values (* *p* < 0.05; ** *p* < 0.01; **** *p* < 0.0001).

To clarify the possible regulatory effects of adiponectin on glucose transport in rat ovarian granulosa cells, adipoRon (the AdipoR1/AdipoR2 dual agonist at 0 μM, 0.1 μM, 1 μM, and 10 μM) was added into the primary cultured granulosa cells. After 24 h of in vitro culture, adding 1 µM of adipoRon significantly enhanced the GLUT1 protein level in rat granulosa cells (*p* < 0.0001) (Figure 2A,B). However, adipoRon had no effects on GLUT3 and GLUT4 protein expression in the rat granulosa cells (Figure 2A,B). Interestingly, 10 µM of adipoRon treatment for 24 h significantly promoted glucose absorption in rat ovarian granulosa cells (Figure 2C).

### 2.3. eCG Administration Significantly Stimulated Ovarian and Blood Adiponectin Levels during the Process of Hormone-Induced Ovarian Growth

**Figure 3:**
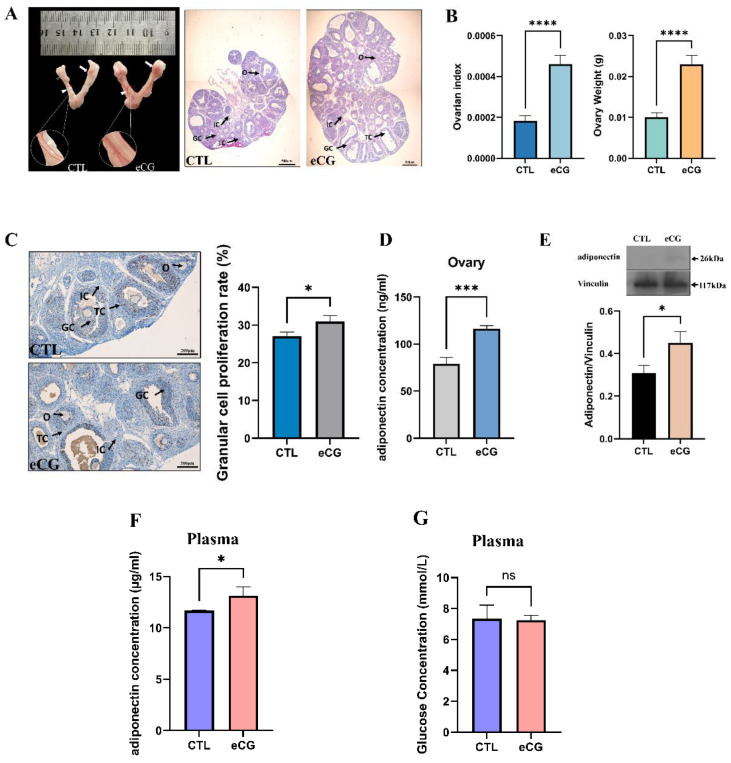
Effects of eCG on ovarian and blood adiponectin secretion during hormone-induced ovarian development in rats. (**A**) The morphology of ovaries and uteri of rats in the eCG-treated and control groups; histological staining of ovaries from eCG-treated and control group rats. (**B**) Ovary weights and ovarian index of rats in the eCG-treated and control groups. (**C**) BrdU staining in rat ovaries from the eCG-treated and control groups and quantitative histograms. (**D**) Concentration of adiponectin in the ovaries of rats by ELISA. (**E**) Ovarian expression of adiponectin in rats from the eCG-treated and control groups. (**F**) Plasma adiponectin levels in rats from the eCG-treated and control groups. (**G**) Plasma glucose levels in rats from the eCG-treated and control groups. Scale bars: 500 μm. The error bars represent means ± SEM (*n* = 3, each group). * Statistically significant values (**** *p* < 0.0001; *** *p* < 0.001; * *p* < 0.05) ns represent no significance. GC, granulosa cell; TC, theca cell; IC, interstitial cell; and O, oocyte.

To confirm the effects of FSH on ovarian adiponectin secretion in vivo, we treated 21-day-old rats with eCG, which is known for its high FSH-like activities in rodents. After 46–48 h of treatment, eCG treatment stimulated ovarian follicular growth, resulting in enlarged ovaries and uterus, more antral follicles, and increased ovarian weight and ovarian index (ovarian weight to body weight ratio) (Figure 3A,B). Furthermore, the BrdU labeling assay revealed prominent nuclear staining in granulosa cells and theca cells of antral follicles, indicating the significantly elevated proliferation rate of granulosa cells after eCG treatment (*p* < 0.05) (Figure 3C).

After 46–48 h eCG of treatment, the ovarian adiponectin protein content was measured by both ELISA and Western blotting. Figure 3D illustrates a significant increase in ovarian adiponectin levels following eCG injection using ELISA. Similarly, the Western blotting results in Figure 3E confirm a significantly higher adiponectin protein expression in the eCG-treated rat ovaries compared to the control group (*p* < 0.05). Simultaneously, eCG administration significantly increased plasma adiponectin levels in rats (*p* < 0.05) (Figure 3F), but with no notable difference observed in plasma glucose levels following eCG injection (Figure 3G).

### 2.4. Positive Correlation between Intraovarian Glucose Transporters and Adiponectin System in eCG-Injected Rats 

**Figure 4:**
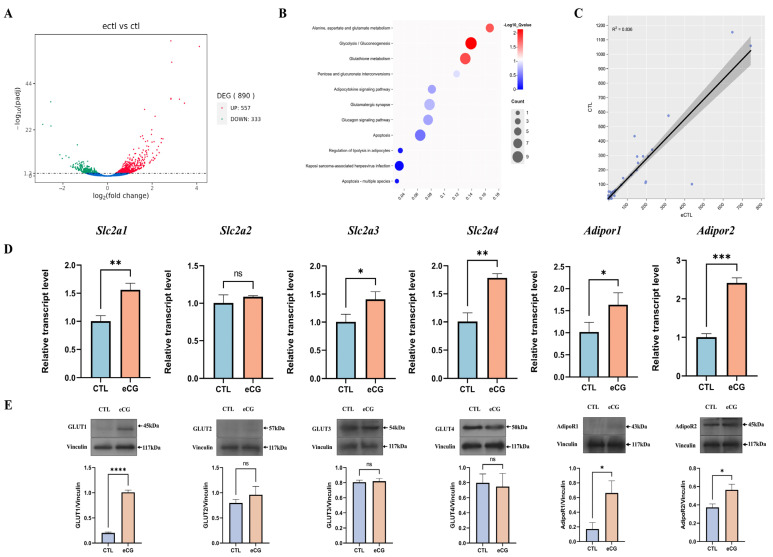
Transcriptome data and correlation analysis of the expression of glucose transporters and the adipokine system in rat ovaries. (**A**) Volcanic map analysis of transcriptome data. Blue: represents no significant difference. (**B**) KEGG pathway analysis; (**C**) correlation analysis of genes related to the glucose metabolism signaling pathway and adipokine signaling pathway. (**D**) Gene transcription levels of *Slc2a1*, *Slc2a2*, *Slc2a3*, *Slc2a4*, *Adipor1*, and *Adipor2* in the ovaries of rats in the eCG-treated and blank control groups. (**E**) Protein levels of GLUT1, GLUT2, GLUT3, GLUT4, AdipoR1, and AdipoR2 in the ovaries of rats in the eCG-treated and blank control groups. The error bars represent means ± SEM (*n* = 3, each group). * Statistically significant values (* *p* < 0.05; ** *p* < 0.01; *** *p* < 0.001; **** *p* < 0.0001); ns represents no significance.

Transcriptome analysis data are shown in Figure 4A–C. Volcano plots revealed 890 differentially expressed genes (DEGs) in rat ovaries post-eCG treatment, including 557 up-regulated and 333 down-regulated genes (Figure 4A). Further KEGG pathway analysis on these differential genes indicated an augmented glucose metabolism and the adipokine signaling pathway after eCG treatment (Figure 4B). Moreover, we conducted a correlation analysis of the enriched genes related to the glucose metabolic pathway and the adipokine signaling pathway, showing how these two signaling pathways have a strong positive correlation with eCG treatment (Figure 4C). Moreover, the relative genes were chosen, and their expressions were verified by real-time quantitative PCR. As shown in Figure 4D, the transcript levels of *Slc2a1*, *Slc2a3*, and *Slc2a4* in rat ovaries were remarkably upregulated after eCG administration (*p* < 0.05). Similarly, *AdipoR1* and *AdipoR2* mRNA expressions in eCG-treated rat ovaries were significantly elevated compared to the control group (*p* < 0.05). In addition, the protein expressions and localizations of those molecules were also measured in rat ovaries. As shown in Figure 4E, the protein expressions of AdipoR1, AdipoR2, and GLUT1 were elevated by eCG treatment, but the ovarian protein expression of GLUT2, GLUT3, and GLUT4 exhibited no significant changes after eCG treatment. 

## 3. Discussion

In the current study, we presented novel findings elucidating that FSH, through the PKA signaling pathway, induced upregulation in adiponectin protein expression in ovarian granulosa cells in vitro. Moreover, the adiponectin receptor agonist (adipoRon) significantly increased GLUT1 protein expression in cultured primary granulosa cells, along with increased glucose absorption. Further in vivo studies using eCG-injected rats confirmed the stimulatory effects of FSH on blood and ovarian adiponectin expression. The subsequent ovarian transcriptome, gene, and protein analysis showed a positive correlation between the expression of intraovarian glucose transporters and the adiponectin system. These findings demonstrated for the first time that FSH regulated adiponectin autocrine secretion via PKA signaling, which might potentially influence the process of glucose transport in ovarian granulosa cells. 

In this study, the positive staining of adiponectin, along with its two receptors, adipoR1 and adipoR2, was detected within ovarian granulosa cells. This finding implies that adiponectin may function as a potential autocrine factor within rat ovaries, although it is widely accepted as an adipose tissue-secreted adipokine. Indeed, numerous studies have established adiponectin as a locally secreted protein in various tissues or cells, including skeletal muscle cells, endothelial cells, osteoblasts, and liver parenchymal cells. However, adiponectin secretion in the ovaries has not been fully elucidated. In prior studies, Chabrolle et al. demonstrated that ovarian adiponectin, adipoR1, and adipoR2 mRNA levels can be regulated by human chorionic gonadotropin [32]. FSH has also been shown to promote ovulation by increasing the production of adiponectin in ovarian follicular fluid [33]. In addition, Tosca et al. showed that the FSH analog (follitropin) significantly increased the mRNA of adipoR1 and adipoR2 in granulosa cells [34]. Consistent with these findings, we employed Western blotting and ELISA assays to demonstrate that FSH stimulated adiponectin secretion in primary cultured granulosa cells within 2 h of culture, providing direct evidence of rapid adiponectin release in ovarian granulosa cells in response to FSH. As a crucial modulator stimulating the activation, growth, and progression of ovarian follicle development, FSH is extensively involved in the intricate interplay of multiple signaling pathways, such as PKA, AKT, and ERK [35,36]. In this study, we demonstrated that FSH primarily enhances adiponectin expression in granulosa cells through the PKA signaling pathway, which is a key component of FSH-mediated cellular metabolism and steroidogenesis [37]. Moreover, this study illustrates that eCG injection upregulated the adiponectin mRNA and protein levels both in rat ovaries and the blood. Given the apparent FSH-like activity of eCG in rodents, these results further confirm the stimulatory effect of FSH on ovarian adiponectin secretion. Altogether, these results confirm that FSH stimulates adiponectin expression in rat ovarian granulosa cells via the PKA signaling pathway, possibly affecting ovarian development and function through autocrine mechanisms. However, we cannot exclude the involvement of another signaling pathway in the FSH-induced expression of adiponectin, which needs further investigation in the future.

In mammals, adiponectin has been observed to affect glucose transport and uptake within different tissues. For instance, adiponectin affects glucose homeostasis in placental tissues by inhibiting GLUT1 expression in early pregnancy in human chorionic villi [38]. In mouse embryos and embryonal carcinoma cells, adiponectin may stimulate glucose uptake through the action of GLUT4 [39]. In skeletal muscle cells, globular adiponectin increases GLUT4 translocation and glucose uptake but decreases glycogen synthesis [40]. In the ovaries, adiponectin is widely reported to be involved in the growth of granulosa cells, steroidogenesis, and follicular development [15,19]. However, its impact on ovarian energy supply or glucose uptake is not fully understood. In this study, we analyzed the transcriptome data of eCG-treated rat ovaries and found significant differences in pathways related to glucose utilization and adipokines among the enriched differentially expressed genes. The following correlation analysis indicated a strong correlation between adipokine signaling pathways and glucose utilization pathways. Previous studies in mammalian ovaries indicate that GLUTs are crucial for maintaining glucose metabolism and providing energy for ovarian function. The present study showed a significant increase in the expression of *Slc2a1*, *Slc2a3*, *Slc2a4*, *adipoR1*, and *adipoR2* genes in rat ovaries after eCG treatment, accompanied by a notable elevation in GLUT1, adiponectin, AdipoR1, and AdipoR2 protein levels. Studies in bovine indicate that GLUT4 might play a supportive role in bovine follicles and corpora lutea growth and glucose uptake [19,41]. In LβT2 gonadotropin-secreting cells, it has been found that GLUT1 expression and translocation to the cell membrane can be regulated by FSH [42]. However, our study does not deny the effect of other facts that influence adiponectin secretion on GLUT expression, as recent studies have elucidated numerous pathways (e.g., DNA methylation, re-modification, non-coding RNA, etc.) that affect gene expression [43,44,45,46]. In rat granulosa cells, FSH has been found to induce glycolytic metabolism through the HIF-1α-AMPK-GLUT1 signaling pathway [20,47]. However, eCG-treated rats exhibited no increase in the circulating glucose levels in this study, which might have been caused by the intricate interactions of metabolic factors in the whole body. For example, a recent study on the pancreatic islet proved that the blockade of FSH signaling or a high FSH level can cause abnormal glucose tolerance due to insufficient insulin secretion [48,49]. Thus, we wondered whether FSH-stimulated adiponectin expression affected glucose utilization in granulosa cells. By adding adipoRon (an adiponectin receptor agonist) in cultured granulosa cells, we proved that adipoRon significantly upregulated glucose uptake and the glucose transporter (GLUT1) protein expression in rat granulosa cells after 24 h of culture. These findings clarify the potential involvement of FSH-stimulated adiponectin in the regulation of glucose uptake in granulosa cells.

**Figure 5:**
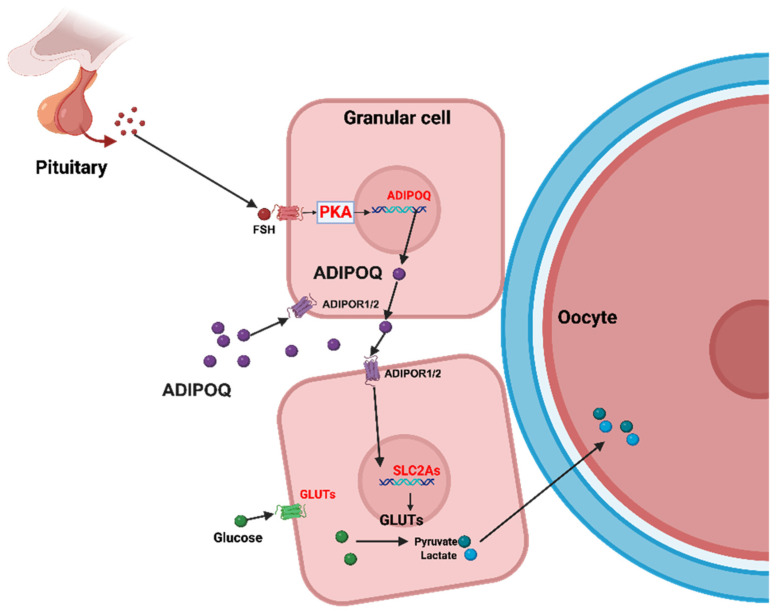
Schematic diagram of the mechanism and role of FSH on adiponectin secretion and glucose transport in ovarian granulosa cells. FSH, follicle-stimulating hormone; PKA, protein kinase A system; SLC2a, solute carrier family 2 member; and GLUTs, facilitative glucose transporters.

This study delineates a specific endocrine regulatory mechanism in rat ovarian granulosa cells, demonstrating that FSH induces the secretion of adiponectin through the PKA signaling pathway (Figure 5). We observed that adiponectin influences the expression of glucose transport proteins, implicating its role in the regulation of follicular development and functionality. These results offer precise insights into adiponectin’s autocrine functions within the ovarian microenvironment, highlighting its significance in glucose transport and utilization—critical factors in follicle energy metabolism and oocyte maturation. Clinically, understanding this relationship provides a valuable framework for developing targeted interventions in treating ovarian dysfunctions and enhancing fertility treatments.

## 4. Methods

### 4.1. Animals

A total of 30 Sprague Dawley rats were purchased from SiPeiFu (Beijing, China) Biotechnology Co. All animals were housed in environmentally controlled cages with a 12 h/12 h light/dark cycle. After acclimatization, the rats aged 21 days (body weight 43.5 ± 2.1 g) were divided into two parts for treatment, one part for in vitro experiments to collect ovarian granulosa cells for the in vitro culture, and the other part for in vivo experiments with 5 IU of eCG injection. A total of 48 h later, plasma samples were collected into heparin-spiked tubes and centrifuged at low speed (3000 rpm for 20 min). SD rats were euthanized with 120 mg/kg pentobarbital sodium (Beijing BioDee Co., Ltd., Beijing, China) [50,51], and their ovaries were dissected. We collected the supernatant (plasma) after centrifugation and stored it at a low temperature (−20 °C). The ovaries were weighed, and their cross-sectional diameters were measured (*n* = 20). One side of the ovaries of SD rats (*n* = 10) was fixed in 4% paraformaldehyde for histological and immunohistochemical analyses, whereas the other side of the ovaries (*n* = 10) was immediately frozen in liquid nitrogen and stored at −80 °C for gene expression analysis. All procedures followed the Animal Care and Use Policy of the Ethics Committee of Beijing Forestry University and were approved (EAWC_BJFU_2023026).

### 4.2. Primary Granulosa Cell Culture In Vitro

The freshly obtained ovaries of 21-day-old rats (*n* = 6) were placed in PBS to remove the perivitelline adipose tissue and oviducts, etc., then incubated in 6 mM of EGTA (Sigma, LOT: SLBG8546V, Milwaukee, WI, USA)-M199 for 5 min, transferred to 0.5 mol/L of sucrose-M199 for 15 min, and then finally transferred to fresh M199 medium (Gibcoo, LOT: 2333381, GrandIsland, NY, USA). After the completion of ovary collection, all in vitro experiments were repeated 3 times. All antral follicles were punctured with a 27-gauge needle under a microscope, releasing granulosa cells and oocytes. The granulosa cells were then blown and mixed, and oocytes and other tissue debris were filtered with a 40 µm cell sieve. The collected granulosa cell suspension was centrifuged at 1000 rpm for 5 min to remove the supernatant. The cells were then resuspended with 1 mL of M199 added with 10% FBS, and then viable cells were counted using the Taipan blue assay. After that, the cell suspension was diluted with the M199 medium according to the experimental requirements and cultured in the six-well plates (Corning, 3335, Corning, NY, USA) within a 37 °C incubator with 5% CO_2_. The next day, after the cells were attached to the wall, the original culture medium was discarded, and the cells were washed three times with the M199 medium, cultured in serum-free M199 medium for at least 24 h, and then treated with AdipoRon according to the experimental design.

### 4.3. Histology

Ovarian tissues soaked in 4% paraformaldehyde for 24 h were removed, washed three times with PBS for five minutes at a time, dehydrated in an alcohol–xylene gradient, and embedded in paraffin. Paraffin blocks were sectioned at 5 μm thickness. The paraffin sheets were laid flat on warm water and picked up with a bonded slide so that the sheet with the tissue was attached to the surface of the slide. The deparaffinized tissue sections were stained with hematoxylin-eosin. The stained sections were subjected to gradient dehydration and sealed with coverslips glued with neutral resin. The overall histomorphology, follicular distribution, and cell type of the prepared tissue sections were observed under the microscope.

### 4.4. Immunohistochemistry

Deparaffinized ovarian sections were incubated in a citrate buffer solution and then blocked with 10% goat serum. The tissues were incubated with primary antibodies including AdipoR1 (sc-46748, Santa Cruz Biotechnology, Dallas, TX, USA), AdipoR2 (sc-46751, Santa Cruz Biotechnology, Santa Cruz, Dallas, TX, USA), Adiponectin (ab181281, Abcam Biotechnology, Cambridge, UK), GLUT1 (21829-1-AP, Proteintech Biotechnology, Wuhan, China), GLUT2 (20436-1-AP. Proteintech Biotechnology, Wuhan, China), GLUT3 (20403-1-AP, Proteintech Biotechnology, China), GLUT4 (sc-53566, Santa Cruz Biotechnology, Dallas, TX, USA), and BrdU (sc-6326, Santa Cruz Biotechnology, Dallas, TX, USA). Tissues were further processed with a goat anti-rabbit IgG/HRP kit (KGOS60, KeyGEN BioTECH Biotechnology, Nanjing, China), stained with the diaminobenzidine solution (30 mg DAB, 150 mL 0.05 M pH 7.6 Tris HCl solution, 25 μL H_2_O_2_) and counterstained with hematoxylin [52]. We scored the positive signals of the DAB staining of ovarian tissue with ImageJ (1.53k, National Institutes of Health, Bethesda, MD, USA) (− represents a negative, +++ represents a strong positive) [53].

### 4.5. Glucose and Adiponectin Assay

The concentrations of adiponectin in rat plasma and adiponectin in rat ovaries were measured using ELISA kits (CSB-E07271r for adiponectin, Cusabio Biotech Co., Wuhan, China) according to the protocol. The data at 450 nm were subsequently read with a microplate reader (PT 3502 g, Beijing Potenov Technology Co., Beijing, China). The intra-assay coefficient of variation was 4.5%, and the inter-assay coefficient of variation was 7.3% in the hormone determination of adiponectin, where ELISA experiments were made by adding 1 mL of 1× PBS per 100 mg of tissue to make a homogenate. Glucose content was determined by the Northern Institute of Biochemistry (Beijing, China). In this experiment, plasma samples and primary granulosa cell samples were obtained from anesthetized rats, and 48 h after eCG injection in the rats, the ovaries were collected for primary granulosa cell culture. The follicles were punctured using a physical method to release the granulosa cells, which were divided into 1 × 10^6^ cell numbers into six-well plates, and the samples were collected 24 h after the addition of adipoRon; 250 µL of cell lysate RIPA were added per 1 × 10^6^ primary granulosa cells.

### 4.6. Western Blot

Ovarian tissues were lysed for protein extraction using the RIPA cell lysate and PMSF, followed by centrifugation (12,000 rpm, 5 min) for the supernatant. All protein extractions were performed on ice, and the protein content of the supernatant (cell lysate) was determined using a BCA protein assay kit. Equal amounts of proteins were separated by SDS-PAGE (12.5%) and transferred to a nitrocellulose membrane. Then, the membrane was placed in a Blocking buffer [ris Buffered Saline + 0.05% Tween 20 (TBS-T) + 5% dehydrated skim milk powder]. The cells were then treated with GLUT1 (1:1000), GLUT2 (1:1000), GLUT3 (1:1000), GLUT4 (1:1000), adiponectin (1:1000), AdipoR1 (1:1000), AdipoR2 (1:1000) and vinculin (1:5000) antibodies before being incubated overnight at 4 °C, washed with TBS-T the next day (6 × 5 min), and then incubated with HRP-labeled secondary antibody (1:5000) for 1 h at room temperature and washed again (3 × 5 min) in TBST. Peroxidase activity was visualized with an ECL kit according to the manufacturer’s instructions and protein content was determined by the densitometric scanning of exposed X-ray films.

The kits and antibodies used in this experiment are listed below: PAGE Gel Fast Preparation Kit 12.5% (PG113, Shanghai Epizyme Biomedical Technology Co., Shanghai, China), SDS-PAGE Electrophoresis Buffer (PM5060, Coolaber Biotechnology, Beijing, China), TBS (T) (PM5080, Coolaber Biotechnology, Beijing, China), and Western transfer buffer (PM5070, Coolaber Biotechnology, Beijing, China). The goat polyclonal anti-rabbit AdipoR2 (sc-46751, Santa Cruz Biotechnology, Dallas, TX, USA), rabbit polyclonal anti-mouse AdipoR1 (sc-46748, Santa Cruz Biotechnology, Dallas, TX, USA), goat polyclonal anti-rabbit GLUT1 (21829-1-AP, Proteintech Biotechnology, Wuhan, China), rabbit polyclonal anti-mouse GLUT2 (20436-1-AP, Proteintech Biotechnology, Wuhan, China), rabbit polyclonal anti-rabbit GLUT3 (20403-1-AP, Proteintech Biotechnology, Wuhan, China), rabbit polyclonal anti-mouse GLUT4 (sc-53566, Santa Cruz Biotechnology, Dallas, TX, USA), rabbit polyclonal anti-mouse vinculin (sc-73614, Santa Cruz Biotechnology, Dallas, TX, USA), and rabbit polyclonal anti-mouse adiponectin (ab181281, abcam Biotechnology, Cambridge, UK) were also used.

### 4.7. Real-Time Quantitative PCR

Total RNA from the ovaries of rats was extracted using the Trizol Kit (Invitrogen, Carlsbad, CA, USA), and the concentration was adjusted to 250 ng/μL. cDNA was synthesized with the StarScript II RT MasterMix, RNA (1000 ng), and random primer (GenStar, Beijing, China). The 10 μL system (3 μL of cDNA, 0.3 μL of forward and reverse primers (100 μg/mL), 5 μL 2× Power SYBR Green PCR master mix, and 1.4 μL ddH_2_O) was configured according to the FastStart DNA MasterPlast SYBR Green Kit (Roche Molecular Systems Inc., Basel, Switzerland) protocol, the primer design of which is shown in Table 2. All these primers were designed using Primer3 software 4.1.0. The data were measured using an ABI PRISM 7500 Fast Real-Time PCR System (Applied Biosystems, Foster City, CA, USA). The performance of the ABI PRISM 7500 Fast Real-Time PCR System we used was in good condition and could reach 100% operation capacity. The specificity and amplification fold of the designed primers were within the confidence interval (15 < CT < 35). The specificity and CT values of the primers we used in the melting curve and amplification curve experiments were within the error range, and the results obtained were credible. The amplification plot and melting curve are shown in Figure S1. Preheating at 95 °C for 10 min was followed by 40 cycles (95 °C for 30 s, 60 °C for 30 s, 72 °C for 30 s) and finally 60–95 °C melt curve progression. The relative expression of target genes was analyzed according to the expression of the internal reference Tubulin. 

### 4.8. Construction of Transcriptome Libraries 

The total RNA (1000 ng) of ovarian tissue (*n* = 3) was extracted following the protocol of the Trizol kit. The mRNAs in the total RNA were enriched by magnetic beads with oligo (DT) and subsequently randomly interrupted. The fragmented mRNA was synthesized as single-stranded cDNA using the M-MuLV reverse transcriptase system, followed by the addition of DNA polymerase I, dNTPs, and a buffer to synthesize stable cDNA double strands. cDNA was purified by the AMPure XP system (Beckman Coulter, Beverly, MA, USA), and 200–250 bp cDNA was selected for PCR amplification. The amplification products were purified again using AMPure XP Beads, resulting in a cDNA library. Transcriptome libraries were sequenced by the Illumina HiSeq platform (Allwegene, Beijing, China).

### 4.9. Transcriptome Analysis

Adapters and low-quality reads were removed from raw reads to obtain clean data. Using the previously described method, the measurement of the gene expression level was obtained [54]. Using the DESeq R package, the differentially expressed genes (DEGs) of two periods were examined and identified. The DEGs were identified using an adjusted *p*-value of 0.05, and the further implementation of the enrichment analyses of DEGs using Gene Ontology (GO) and Kyoto Encyclopedia of Genes and Genomes (KEGG) was performed with KOBAS software (version 3.0, accessed on 1 January 2023) [55].

### 4.10. Immunocytofluorescence

Granulosa cells were initially inoculated into 35 mm dishes with coverslips and subsequently washed with PBS for 5 min. Following this, 4% paraformaldehyde (PFA) was added dropwise onto the cell cultures and left to incubate at room temperature for 40 min, which was then washed thrice with PBS for 5 min each time. Permeabilization was achieved by adding 0.25% Triton X-100 dropwise onto the cell cultures, incubating at room temperature for 10 min, and washing thrice with PBS for 5 min each. The cell slide was then treated by applying donkey serum dropwise, which was kept closed at room temperature for 1 h. After the removal of the donkey serum, the primary antibody solution was added dropwise onto the samples, and the antibody dilution solution was similarly applied to the negative control. Subsequently, the samples were refrigerated at 4 °C overnight, brought back to room temperature the following day, and washed thrice with PBS buffer for 5 min each time. Next, the cell slide was placed in a light-proof wet box, and the fluorescent secondary antibody solution was added dropwise, followed by incubation for 1 min at room temperature in a light-proof box. Following incubation, cells underwent an additional 1 h of incubation at room temperature and were washed thrice with PBS for 5 min each. The staining with DAPI solution lasted for 1 min. Finally, the stained cell cultures were mounted onto slides for observation and imaging under a fluorescence microscope.

### 4.11. Statistical Analysis

As detailed in the figure legends, the results are presented as the means ± SEM of at least three independent experiments. All data were subjected to one-way (repeated measure) ANOVA (GraphPad Prism Software 9.0, Inc., San Diego, CA, USA). Tukey’s test determined significant differences between the treatment groups. Statistical significance was inferred at *p* < 0.05.

## 5. Limitations of This Study

The regulatory role of FSH on adiponectin secretion in ovarian granulosa cells and its subsequent impact on the expression of glucose transporters and glucose uptake has been elucidated by our study. However, it has not yet been established whether the modulation of glucose transporter expression and glucose uptake by FSH occurs specifically through adiponectin expression. This particular aspect remains to be explored and necessitates further investigation.

## Figures and Tables

**Table 1:** Immunolocalization of adiponectin in the ovaries of rats.

Antibody	GC		TC		IC		O	
	NC	eCG	NC	eCG	NC	eCG	NC	eCG
Adiponectin	−	+++	−	+	−	++	−	++

Immunohistochemical staining was determined as negative (−), positive (+), strongly positive (++), and very strongly positive (+++). Staining that was weak but higher than that of the control was set as positive (+). The highest intensity of staining was set as very strongly positive (+++). A staining intensity between + and +++ was set as strongly positive (++). No signal was set as negative (−). eCG, equine chorionic gonadotropin; NC: negative control; GC, granular cell; TC, theca cells; IC, interstitial cell; and O, oocyte.

**Table 2:** Primer sequences used for mRNA RT-PCR.

Accession Number	Gene Symbol	Primer Forward (5′-3′)	Primer Reverse (5′-3′)	Product Length
NM_138827	*Slc2a1*	AGGCCCTGGTCCTATTCCAT	CTTGTCACTTTGGCTGGCAC	289
NM_012879	*Slc2a2*	TCATGTCGGTGGGACTTGTG	ACACGTAAGGCCCAAGGAAG	252
NM_001412552	*Slc2a* *3*	CAGCTCCAGCAAGCAATTCG	AGCTACCTCAAACACACCCG	137
NM_012751	*Slc2a* *4*	GGCTCTGACGTAAGGATGGG	AGTGTTCCAGTCACTCGCTG	52
NM_207587	*Adipor1*	GTCCTGGTGGTGGCAGCGGCTT	CGGCCCGGAGGCTGTGCCAGTG	266
NM_001037979	*Adipor2*	GACGGGCAACATTTGGACAC	AAAGGCAGAGAATGGCTCCC	243
NM_022298	*Tubulin*	GACTCGTCGTACTCCTGCTT	AAGACCTCTATGCCAACACC	131

## Data Availability

The datasets used and/or analyzed during the current study are available from the corresponding author upon reasonable request.

## Supplementary Materials

The following supporting information can be downloaded at: https://www.mdpi.com/article/10.3390/ijms25105155/s1.

## Abbreviations

FSH	follicle-stimulating hormone
PKA	protein kinase A system
GLUTs	facilitative glucose transporters
SLC2a	solute carrier family 2 member
HIF	hypoxia inducible factor
AMPK	adenosine 5′-monophosphate (AMP)-activated protein kinase
DAPI	4′,6-diamidino-2-phenylindole
DMSO	dimethyl sulfoxide
H89	dihydrochloride
FSK	Forskolin
TC	theca cell
GC	granulosa cell
IC	interstitial cell
O	oocyte
eCG	equine chorionic gonadotropin
AKT	protein kinase B
ERK	extracellular signal-regulated kinase
