# Supplementary material for: Regulation and Role of Adiponectin Secretion in Rat Ovarian Granulosa Cells
Source: Int J Mol Sci. 2024 May 9;25(10):5155. doi: 10.3390/ijms25105155 (PMC11120769; doi:10.3390/ijms25105155)
Supplement: Supplementary file 1 [file ijms-25-05155-s001.zip › ijms-2962982-supplementary.pdf]

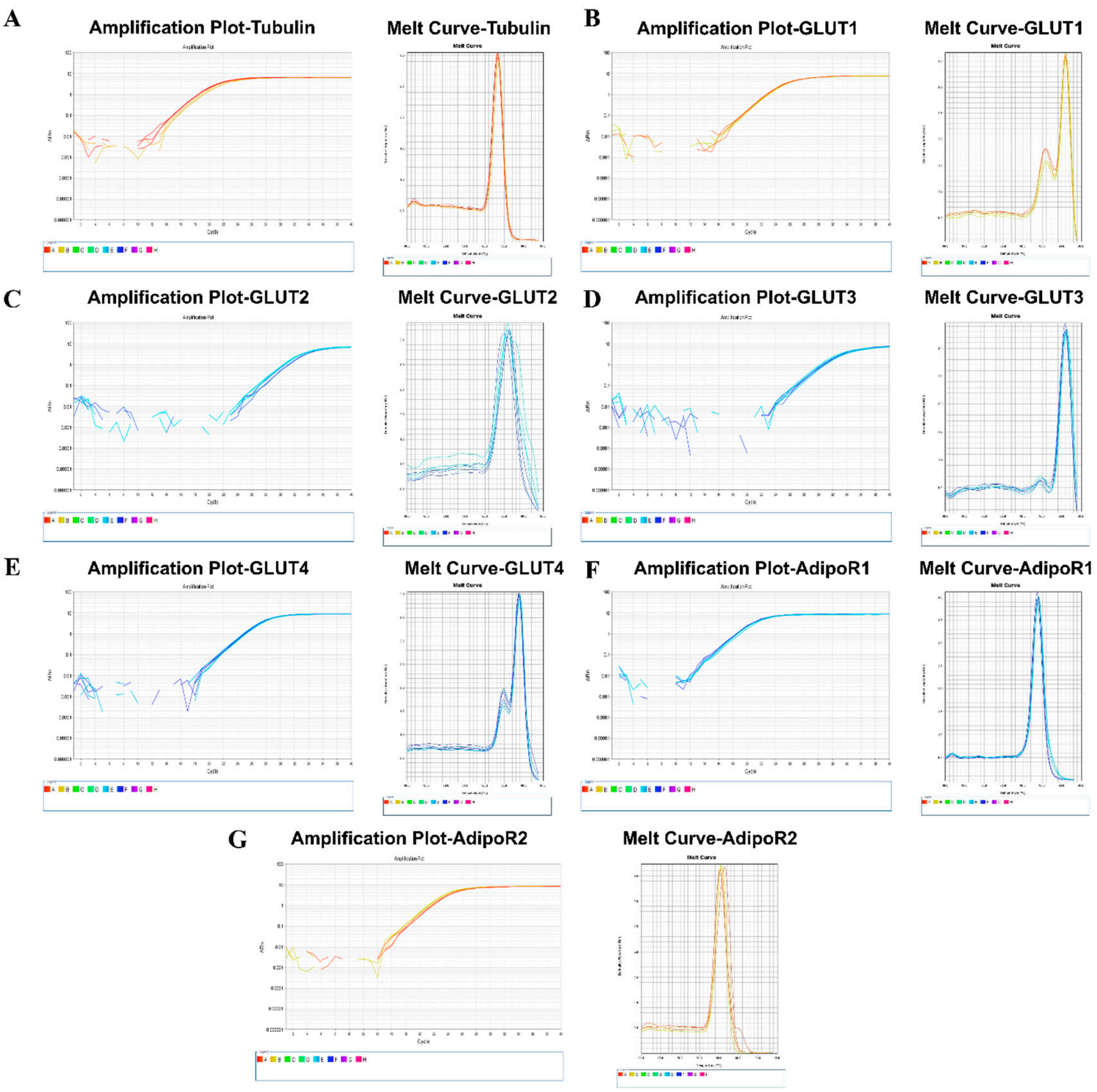

Figure S1. The PCR amplification curve and melting curve of each gene used in the experiment. (A-H) are the amplification curves and melting curves of GLUT1-4, AdipoR1, 2.
